# Cobalt Encapsulated in Nitrogen-Doped Graphite-like Shells as Efficient Catalyst for Selective Oxidation of Arylalkanes

**DOI:** 10.3390/molecules29010065

**Published:** 2023-12-21

**Authors:** Shuo Li, Shafqat Ali, Zareen Zuhra, Huahuai Shen, Jiaxiang Qiu, Yanbin Zeng, Ke Zheng, Xiaoxia Wang, Guanqun Xie, Shujiang Ding

**Affiliations:** 1 School of Materials Science and Engineering, Dongguan University of Technology, Dongguan 523808, China; jingtao360@126.com (S.L.); shenhh2020@126.com (H.S.); 2019119@dgut.edu.cn (K.Z.); 2 School of Chemistry, Xi’an Key Laboratory of Sustainable Energy Materials Chemistry, Xi’an Jiaotong University, Xi’an 710049, China; dings@mail.xjtu.edu.cn; 3 School of Environment and Civil Engineering, Dongguan University of Technology, Dongguan 523808, China; aliqau22@gmail.com (S.A.); zareenzuhraqau@yahoo.com (Z.Z.); qiujx1016@126.com (J.Q.); 15089498680@163.com (Y.Z.)

**Keywords:** selective oxidation, metallic cobalt, N-doped graphite-like carbon, core-shell structure, synergistic effect

## Abstract

Selective oxidation of ethylbenzene to acetophenne is an important process in both organic synthesis and fine chemicals diligence. The cobalt-based catalysts combined with nitrogen-doped carbon have received great attention in ethylbenzene (EB) oxidation. Here, a series of cobalt catalysts with metallic cobalt nanoparticles (NPs) encapsulated in nitrogen-doped graphite-like carbon shells (Co@NC) have been constructed through the one-pot pyrolysis method in the presence of different nitrogen-containing compounds (urea, dicyandiamide and melamine), and their catalytic performance in solvent-free oxidation of EB with tert-butyl hydrogen peroxide (TBHP) as an oxidant was investigated. Under optimized conditions, the UCo@NC (urea as nitrogen source) could afford 95.2% conversion of EB and 96.0% selectivity to acetophenone, and the substrate scalability was remarkable. Kinetics show that UCo@NC contributes to EB oxidation with an apparent activation energy of 32.3 kJ/mol. The synergistic effect between metallic cobalt NPs and nitrogen-doped graphite-like carbon layers was obviously observed and, especially, the graphitic N species plays a key role during the oxidation reaction. The structure–performance relationship illustrated that EB oxidation was a free radical reaction through 1-phenylethanol as an intermediate, and the possible reaction mechanistic has been proposed.

## 1. Introduction

The selective oxidation of arylalkanes to desired oxygen-containing compounds is a valuable reaction in the chemical industry with the increasing demand for various functional molecules by modern society. A representative example is the selective oxidation of ethylbenzene to acetophenone, which is an important organic intermediate for various pharmaceuticals, perfumes and fine chemicals [1,2,3,4,5,6,7,8]. However, the available methods for ethylbenzene oxidation generally show some drawbacks, such as harsh reaction conditions (e.g., high temperature/pressure), environmental pollutions (e.g., hazardous oxidants and organic solvents) and low catalytic performances [9,10,11,12]. As a result, it is interesting and urgent to solve these problems in the basic research and industrial practice of EB oxidation. In this regard, development of novel and efficient heterogeneous catalysts for EB oxidation is still highly desirable.

Nitrogen-doped carbo-catalysts (NCs) have shown promising ability in the selective oxidation of ethylbenzene to acetophenone as a kind of metal-free catalyst [4,6,13,14,15]. For example, Wang et al. [15] prepared a series of nitrogen doped carbon materials without metal as catalyst for solvent-free EB oxidation, where the graphitic N species and meso-/macro-porous structure were proposed as contributors to the high catalytic activity. However, the efficiency of the NCs was rather unsatisfactory. It should be noted that NCs combined with various metals have been found to significantly improve catalytic activities in selective hydrogenation [16,17,18,19], CO_2_ fixation [20,21,22] and electrocatalysis [23,24,25]. A similar phenomenon could be observed in the field of C-H bonds oxidation. NCs combined with cobalt (Co/NCs) have been prepared by a variety of methods and investigated as catalysts for arylalkane oxidation with various oxidants [26,27,28,29,30,31,32,33]. For instance, cobalt single atoms supported on carbon nitride (Co SACs) showed 46% conversion of ethylbenzene using air as oxidant, whereas the nanosized or homogenous Co catalysts were surprisingly inert to the reaction [28]. Very recently, Hosseini [9] reported a Co/N-CFF@TiO_2_-SiO_2_ catalyst composed of cobalt oxide incorporated in N-doped carbon and wrapped in a TiO_2_-SiO_2_ layer for EB oxidation in oxygen. The EB conversion and acetophenone selectivity reached 25% and 88% under optimized aerobic conditions (130 °C, 2 Mpa and 12 h), respectively.

Although the above examples have illustrated that the Co/NC catalysts display remarkable catalytic performance for EB oxidation, the synthesis process of these catalysts is tedious or complicated and may involve high-cost Co/N sources or harsh preparation conditions [3,9,27,29,30,31,32,33,34,35,36,37,38]. Wang [34] designed composite catalysts of N-doped carbon with cobalt and gold for selective oxidation of alcohol into ester. However, the complicated process and high cost of synthesizing MOFs is not in line with the principles of green and sustainable chemistry. Liu [3] synthesized a Co/N co-doped nanoreactor with high-cost cobalt porphyrin as precursor to construct Co-N_x_ sites for EB oxidation. Unfortunately, the synthesis method of the catalyst was very complicated and requires a high temperature (800 °C) treatment in N_2_. In another similar study, the Co_3_O_4_@PNC-400 catalyst has a synergistic effect between Co_3_O_4_ and the nitrogen-carbon framework during C-H oxidation by using cobalt porphyrin as Co/N source for the catalyst preparation [38]. On the other hand, we know that cobalt species have many crystalline phases (e.g., Co_3_O_4_, CoO, Co^0^), it is difficult to obtain a single cobalt crystalline phase in the catalysts for EB oxidation, and this is not conducive to research into the oxidation mechanism [31,32,39]. Such drawbacks in Co/NC catalyst preparation restrain developments and practical applications. Therefore, a simple, effective and affordable method to prepare Co/NCs catalysts is of great significance for EB oxidation.

In the present study, we use different nitrogen sources and a one-pot pyrolysis method to prepare cobalt-based catalysts with a core-shell nanostructure, leading towards highly efficient selective oxidation of EB to acetophenone. The doped nitrogen atoms in catalysts are introduced from the carbonization of urea, dicyandiamide and melamine. To our knowledge, this is the first report investigating the effects of different nitrogen sources on the construction of Co-based core-shell catalysts for EB oxidation. It was found that the UCo@NC catalyst has the best catalytic activity for EB oxidation in comparison to other catalysts. The effect of different nitrogen sources on the physical and chemical properties of the obtained cobalt-based catalysts were investigated. In addition, the structure–performance relationship of the obtained cobalt-based catalysts was explored in order to clarify the reason for their excellent selective oxidation, and the mechanism of EB oxidation was also probed.

## 2. Results and Discussion

### 2.1. Catalyst Characterization

**Figure 1:**
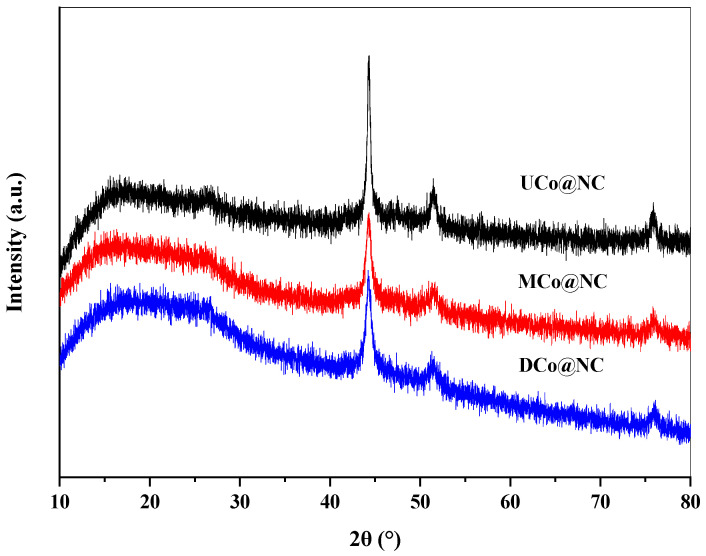
XRD patterns of the as prepared catalysts.

The crystalline structure of the calcined samples was investigated using X-ray diffraction, and the results are displayed in Figure 1. An amorphous peak in the 2θ range of 20° and 30° was attributed to the (002) plane of the graphitic-type lattice [40], and three diffraction peaks at 44.2, 51.5, and 75.9° were attributed to the (111), (200), and (220) crystal planes of the metallic Co^0^ (JCPDS 15-0806). Based on the Scherer equation, the average crystalline size of metallic Co^0^ NPs for the UCo@NC, DCo@NC and MCo@NC catalysts was 20.7 nm, 12.7 nm and 11.0 nm (Table 1), respectively. The cobalt particle size for the UCo@NC was about twice as large as that of the other catalysts. Moreover, the Co@C catalyst without nitrogen source in the precursor does not lead to the sintering of metallic cobalt NPs compared with the DCo@NC and MCo@NC catalysts (Figure S1 and Table 1). These results clearly show that the one-pot pyrolysis method was able to obtain the metallic cobalt NPs directly in the presence of different nitrogen sources, while adding nitrogen sources to the precursor was unfavorable for the formation of smaller metallic cobalt NPs (˂10.9 nm).

**Figure 2:**
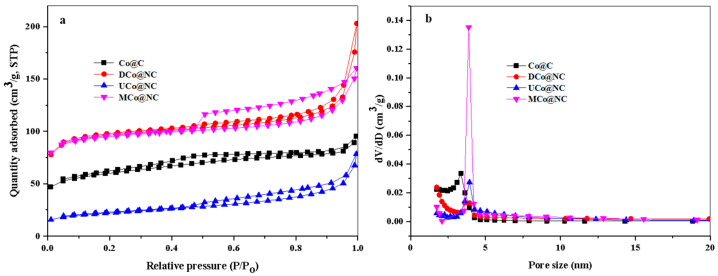
N_2_ adsorption/desorption isotherms (**a**) and pore size distribution curves (**b**) of Co@C, UCo@NC, DCo@NC and MCo@NC.

In Figure 2, N_2_ adsorption/desorption isotherms were acquired in order to examine the textural characteristics of the calcined samples. The catalysts in question exhibit type IV adsorption isotherms accompanied by type H4 hysteresis loops, as classified by IUPAC (Figure 2a). This characteristic suggests the existence of mesoporous nature [41], as was further confirmed by the pore diameter distributions (Figure 2b). Based on the sorption isotherms, the S_BET_, V and D of the catalysts were obtained and summarized in Table 1. The DCo@NC and MCo@NC catalysts displayed a similar specific surface area. As compared with the Co@C catalyst, the introduction of the nitrogen sources markedly increases the specific surface area of the DCo@NC and MCo@NC catalysts. However, it was surprising to find that UCo@NC had lower S_BET_ but larger V values than those of the Co@C catalyst. The moderate carbonization temperature (600 °C) employed here may have facilitated the formation of a mesoporous structure for the samples, thus being helpful to the selective oxidation reaction.

**Figure 3:**
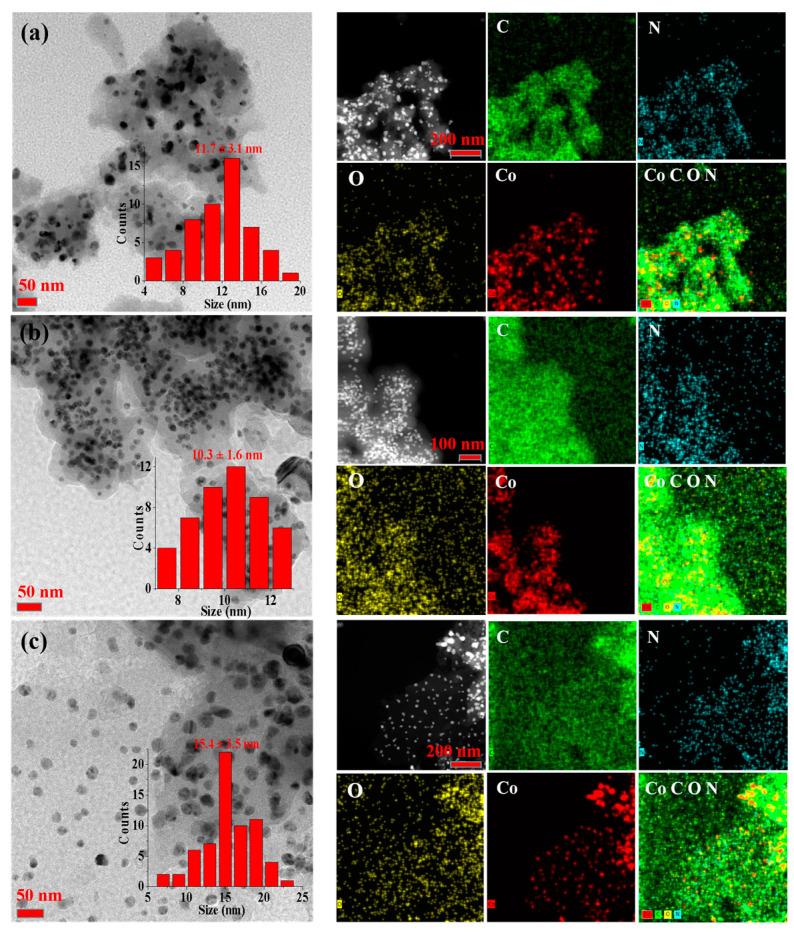
TEM images of (**a**) DCo@NC, (**b**) UCo@NC and (**c**) MCo@NC and the respective element mapping. The insets in Figure 3a–c show the corresponding particle size distributions of Co NPs.

**Figure 4:**
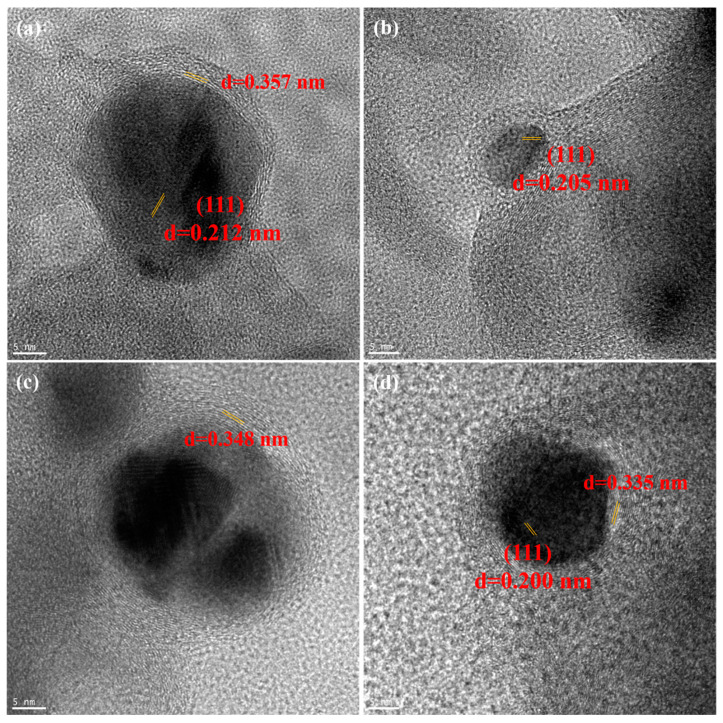
HRTEM images of (**a**) DCo@NC, (**b**,**c**) UCo@NC and (**d**) MCo@NC.

The morphology and nanostructure of the samples were determined by TEM. The TEM images of the Co@NC catalysts are displayed in Figure 3. It was quite clear that the spherical-like cobalt NPs of the three catalysts were dispersed uniformly and no significant aggregates were observed. The average Co particle sizes for the Co@C (Figure S2a), DCo@NC, UCo@NC and MCo@NC were 12.2 nm, 11.7 nm, 10.3 nm and 15.4 nm, respectively. Such results were basically consistent with the calculated values from the Scherer equation except for the UCo@NC sample, which may be the reason that the TEM is a local area characterization method. In addition, the dark-field TEM and corresponding mapping images of the catalysts are also shown in Figure 3. The bright particles in similar sizes were cobalt NPs, and elemental mapping analysis showed the homogeneous dispersion of C, N, O and Co in the samples, indicating the successful doping of the nitrogen species. Furthermore, the high-resolution TEM (HRTEM) images of the catalysts are shown in Figure 4, which demonstrated clearly that the spherical-like particles were well encapsulated in the multilayer graphite-like carbon to form the core-shell nanostructure. The crystal lattices of these core particles are 0.212 nm (Figure 4a), 0.205 nm (Figure 4b) and 0.200 nm (Figure 4d), corresponding to the (111) interplanar spacing of metallic cobalt (Table 1) [42]. The well-ordered carbon layers with a typical interlayer spacing of 0.34 nm belonged to the graphite-like carbon layers [40,43,44,45]. Moreover, a similar core-shell nanostructure was formed in the Co@C prepared in the absence of a nitrogen source (Figure S2b). These TEM results showed that Co@NC catalysts with a metallic cobalt NP core and a nitrogen-doped graphite-like carbon layer shell have been successfully synthesized by the one-pot pyrolysis method. The carbon layers were proposed not only to prevent the aggregation of the metallic cobalt NPs, but also to protect the metallic cobalt from air oxidation, thereby improving the catalytic performance.

**Figure 5:**
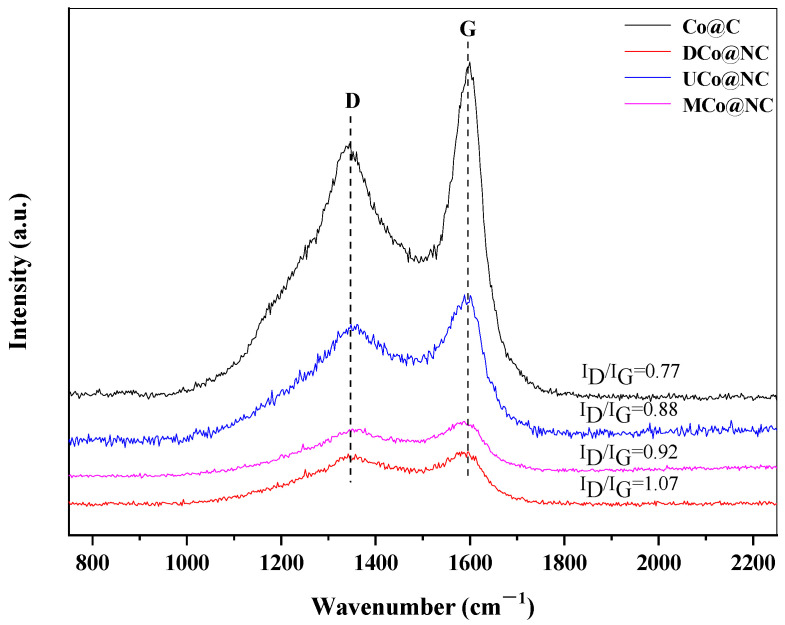
Raman spectra of the as prepared catalysts.

In order to examine the defects in the structure of the acquired catalysts, Raman spectra were acquired and are presented in Figure 5. The spectra of each sample revealed two distinct characteristic peaks at approximately 1360 and 1590 cm^−1^, which corresponded well with the typical Raman modes of the D and G bands representing carbon species, respectively. These peaks indicated the presence of graphite-like carbon within the catalysts [45]. The former was related to the lattice defects, the latter to the in-plane stretching vibration of sp^2^ carbon atoms [46]. The intensity ratio of the D band to G band (I_D_/I_G_) can reflect the formation of defect density, the high I_D_/I_G_ value demonstrating that a large number of N atoms were doped in the carbon layers [43]. The I_D_/I_G_ values of Co@C, UCo@NC, MCo@NC and DCo@NC were 0.77, 0.88, 0.92 and 1.07, respectively. It was found that the I_D_/I_G_ values of the N-doped catalysts were higher than that of Co@C, suggesting that the presence of N atoms in the carbon layers should have promoted the generation of more defects and would be beneficial to enhance the catalytic performance [47]. According to the above characterization results, the synthesis of metallic cobalt NPs encapsulated in N-doped graphite-like carbon shells has been achieved by the one-pot pyrolysis method without a complicated process, and especially the core-shell nanostructures were constructed highly conveniently from low-cost Co/N sources.

**Figure 6:**
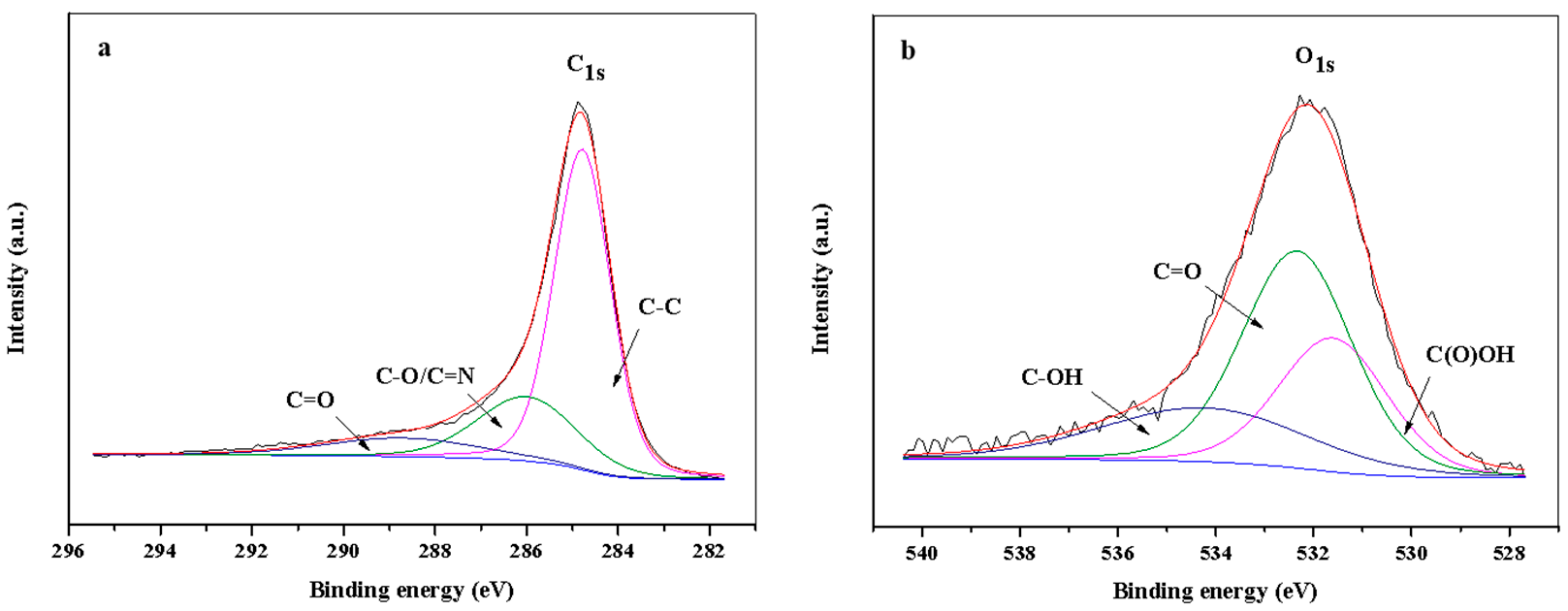
High resolution C_1s_ (**a**) and O_1s_ (**b**) XPS spectra of the UCo@NC catalyst.

Figure S3 shows the full survey spectra of the UCo@NC, DCo@NC and MCo@NC catalysts, which reveals the existence of C, N, O and Co in all the catalysts. Table 2 reports the surface atomic percentage of various elements in different catalysts. Compared with the Co@C (3 at.%) catalyst, the cobalt contents decreased significantly after the introduction of nitrogen sources and varied from 1.1 to 1.2 at.% among the N-doped catalysts, according to the XPS results. The nitrogen contents were 7.3, 8.9 and 9.1 at.% for UCo@NC, DCo@NC and MCo@NC catalysts, respectively. These results apparently suggest that the UCo@NC catalyst has the lowest surface atomic percentage of nitrogen; however, DCo@NC and MCo@NC catalysts have similar nitrogen contents. The high resolution C_1s_ XPS spectra (Figure 6a) of UCo@NC displays three obvious peaks at 284.8, 286.0 and 288.7 eV and these were attributed to the C-C, C-O/C=N and C=O bonds, respectively [45,48], indicating the existence of carbon atoms connected to N and O species. Figure 6b exhibited the O_1s_ spectrum of UCo@NC, which was deconvoluted into three peaks with a binding energy of 531.6, 532.3 and 534.2 eV, corresponding to C(O)OH, C=O and C-OH, respectively [14,32].

**Figure 7:**
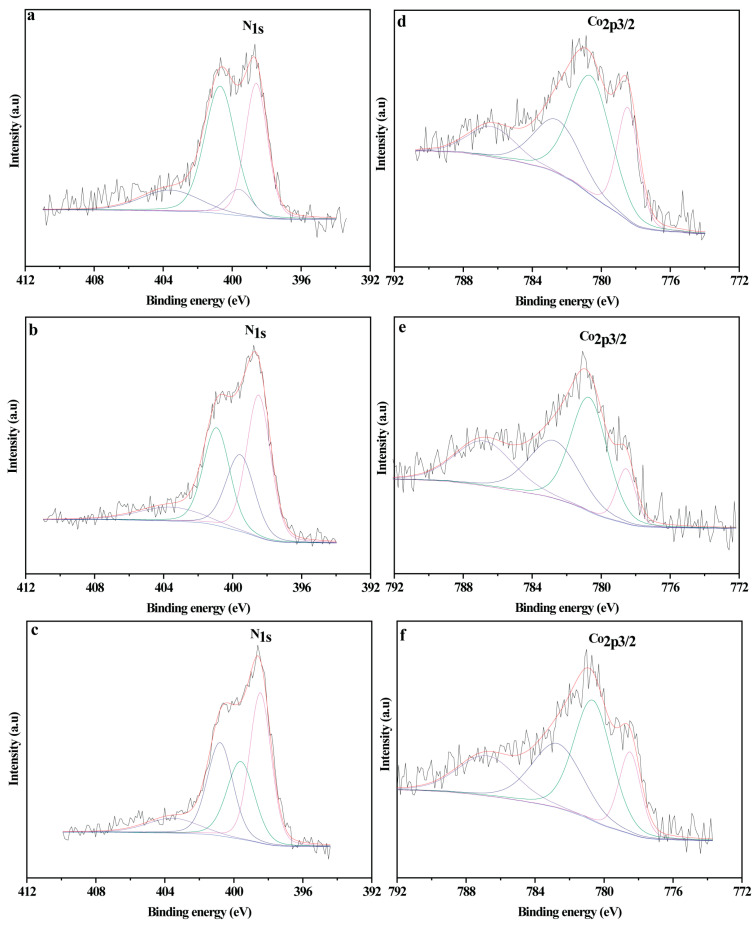
N_1s_ (**a**–**c**) and Co_2p3/2_ (**d**–**f**) XPS spectra of (**a**,**d**) UCo@NC, (**b**,**e**) DCo@NC and (**c**,**f**) MCo@NC catalysts.

The N_1s_ and Co_2p_ XPS spectra of the prepared catalysts were recorded and the results are shown in Figure 7. The high resolution N_1s_ XPS spectrum (Figure 7a–c) displayed four deconvoluted peaks with a binding energy of 398.6, 399.6, 400.8 and 403.6 eV, which were identified as pyridinic N (N1), pyrrolic N (N2), graphitic N (N3) and oxidized N (N4) species, respectively [33,45]. The four types of nitrogen species are shown in Figure S4. Among these catalysts, UCo@NC had the lowest surface atomic percentage of nitrogen (7.3%, Table 2). After fitting analysis of the N_1s_ spectra, the graphitic nitrogen species was highest in the UCo@NC catalyst, while the sum of pyridinic nitrogen and graphitic nitrogen species showed similar values for DCo@NC and MCo@NC catalysts (Figure S5). Different decomposition rates of the nitrogen sources would have different carbonization rates, and exhibit different reducing abilities, which would affect the types of nitrogen species doped in the graphite-like layers. Previous studies have shown that the graphitic nitrogen species was the most active species for the catalyzing oxidation of alkanes [6,14,15]. Moreover, it was reported that the pyridinic and graphitic nitrogen species could combine with cobalt NPs to form a Co-N bond, highly beneficial in improving oxidation reactions [30]. Thus, our work showed that urea could serve as an ideal nitrogen source to increase the nitrogen doping level of Co@NC catalysts, which was expected to boost the catalytic activities for EB oxidation. The Co_2p3/2_ region (Figure 7d–f) showed four peaks for these catalysts, which can be assigned to the Co^0^ species (778.6 eV, 778.5 eV, 778.5 eV), Co^3+^ species (780.7 eV, 780.6 eV, 780.7 eV), and Co^2+^ species (782.7 eV, 782.6 eV, 782.7 eV) and the satellite peaks (786.8 eV, 786.4 eV, 786.8 eV), consistent with previous publications [10,33,49]. The results further confirmed that the metallic cobalt NPs were synthesized successfully through pyrolysis reduction at a moderate temperature in nitrogen. The two/three-valent cobalt species should have been formed by the oxidation of surface Co NPs, since the XPS is a surface analysis technology and the defects in carbon layers allow oxygen to penetrate the shell into the surface Co particles [17]. Lin et al. reported that the metallic cobalt NPs donated an electron to form a positively charged cobalt ion species during EB oxidation [33]. It was interesting to observe that the Co@C catalyst had the highest content of the metallic cobalt species and the values decreased in the order of Co@C > UCo@NC > MCo@NC > DCo@NC (Table 2). Different nitrogen sources could undergo different carbonization mechanisms and produce different numbers of small organic molecules during the calcination process and affect the content of the metallic cobalt species.

Thus, the present simple and low-cost method can afford the desired core-shell nanostructure, with metallic cobalt NPs as core and N-doped graphite-like carbon layers as shell. The N-containing compounds (urea, melamine and dicyandiamide) in the precursors affected the types of nitrogen species and also the content of the metallic cobalt NPs. It was found that urea as nitrogen source was able to form the highest number of graphitic N and metallic cobalt species (Table 2), although with the largest average crystalline size of metallic cobalt NPs (Table 1). Moreover, it was found that the sum of the graphitic and pyridinic nitrogen species between DCo@NC and MCo@NC was similar and higher than that of the UCo@NC catalyst (Figure S5).

### 2.2. Selective Oxidation Performance

**Scheme 1:**
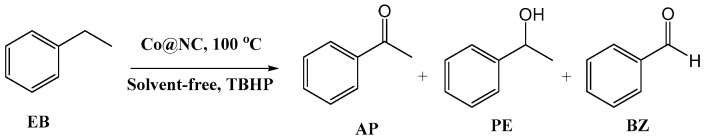
Oxidation of ethylbenzene over Co@NC series catalysts.

The unique nanostructure and morphology of the obtained Co@NC series catalysts inspired us to study their catalytic performance for selective oxidation, where solvent-free oxidation of EB with TBHP as oxidant was used as the probe reaction. The major product was acetophenone (AP), which was contaminated with 1-phenylethanol (PE) and benzaldehyde (BZ) as byproducts (Scheme 1), as identified by GC-MS (Figure S6).

**Figure 8:**
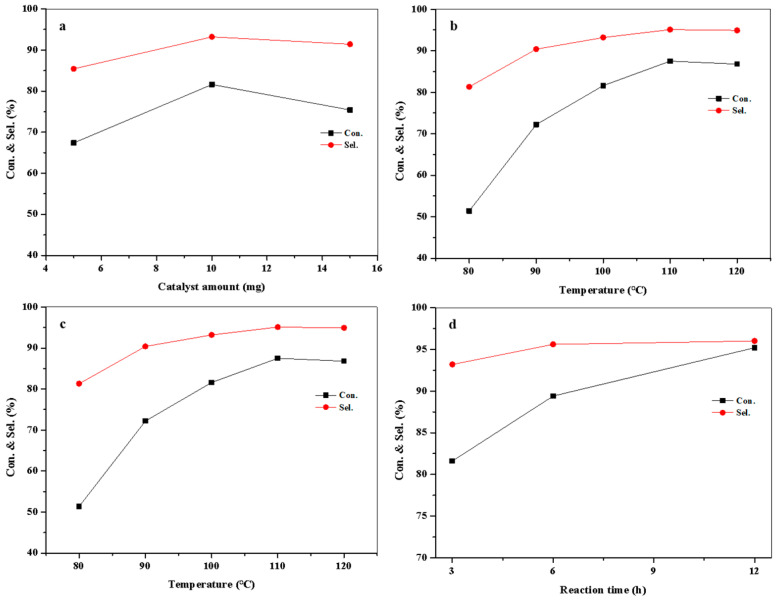
Effect of different parameters on EB oxidation over UCo@NC catalyst. The reactions were performed with 2 mmol EB. (**a**) Catalyst amount optimization under 100 °C with 6 mmol TBHP in 3 h; (**b**) molar ratios of TBHP to EB optimization under 100 °C with 10 mg catalyst in 3 h; (**c**) reaction temperature optimization with 10 mg catalyst and 6 mmol TBHP in 3 h; (**d**) reaction time optimization under 100 °C with 10 mg catalyst and 6 mmol TBHP.

Various reaction parameters were studied in order to fabricate Co@NC series catalysts with high catalytic performance. Figure 8 shows the effects of EB oxidation with different reaction parameters. With the increase in catalyst amount, molar ratios of TBHP to EB and reaction temperature, the conversion and selectivity were improved gradually. When the molar ratio of TBHP to EB was 3 and 10 mg of catalyst was employed, up to 81.6% conversion of EB and 93.2% selectivity of AP were obtained at 100 °C in 3 h. As shown in Figure 8c, both the conversion and selectivity increased slightly when elevating the reaction temperature to 110 °C. However, the TBHP decomposed easily at such a high temperature, and it was unfavorable to prolong the reaction time, since it would cost too much TBHP. Therefore, 100 °C was selected for high conversion and selectivity in the EB oxidation. The influence of reaction time on conversion and selectivity was studied in the range of 3 to 12 h and the results are depicted in Figure 8d; EB could convert to AP with a conversion of 95.2% and selectivity of 96.0% within 12 h. Therefore, 12 h was selected as the optimum reaction time. According to the above experimental results of the UCo@NC catalyst for EB oxidation, the optimum reaction conditions were as follows: 2 mmol of EB, 10 mg of catalyst, TBHP to EB molar ratio of 3, and 100 °C for 12 h under solvent-free conditions.

**Figure 9:**
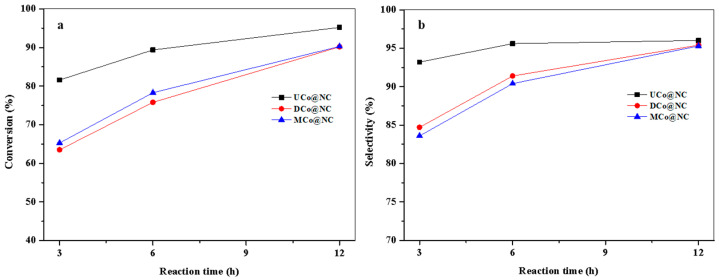
EB conversion (**a**) and AP selectivity (**b**) for the UCo@NC, DCo@NC and MCo@NC catalysts. Reaction conditions: 2 mmol EB; 10 mg catalyst; 3 molar ratio of TBHP/EB; 100 °C.

Table 3 shows the selective oxidation of EB over the different catalysts under optimum reaction conditions. The blank test without catalyst gave only trace EB conversion (Table 3, entry 1), while NC showed 22.9% conversion of EB and 68.3% selectivity to AP (Table 3, entry 2). The Co@C catalyst afforded EB conversion of 55.0% which was higher than that of NC, suggesting that the metallic cobalt is the active site and play a more critical role than NC in EB oxidation. The incorporation of the nitrogen and cobalt species can further boost the oxidation performance, as demonstrated by the Co@NC series catalysts (Table 3, entry 4–6). The improved oxidation performance of the Co@NC series catalysts could be attributed to the synergistic effect of the metallic Co NPs and N-doped graphite-like carbon shells. Notably, an EB conversion of 81.6% and 93.2% selectivity to AP were achieved over UCo@NC catalyst in 3 h (Table 3, entry 5). The UCo@NC catalyst afforded the most excellent catalytic performance, with 95.2% EB conversion and 96.0% AP selectivity when the reaction time was prolonged to 12 h, which was higher than that of DCo@NC and MCo@NC catalysts (Table 3, entry 7–9). In addition, when milder reaction temperatures were carried out, 90.6% EB conversion and 95.8% AP selectivity could be obtained even at 80 °C in 24 h (Table 3, entry 10 and 11). Figure 9 shows the selective oxidation of EB against time over the Co@NC series catalysts. It is interesting to observe that the DCo@NC catalyst has a similar catalytic performance to the MCo@NC catalyst with the prolonging of reaction time. In contrast, the UCo@NC catalyst shows higher EB conversion and AP selectivity than the DCo@NC and MCo@NC catalysts. It is worth noting that both DCo@NC and MCo@NC catalysts exhibited EB conversion of more than 90% and AP selectivity of more than 95% after a reaction time of 12 h. These results clearly suggest that the prepared Co@NC series catalysts showed excellent activity for EB oxidation, among which the UCo@NC catalyst exhibited the best performance for selective oxidation.

The superior conversion and selectivity for EB oxidation shown by the UCo@NC catalyst could be ascribed to the compositional and structural characterization. Since the UCo@NC catalyst had the lowest specific surface area and the largest average cobalt particle size among the three samples (Table 1), which is unfavorable to catalytic activity, other factors should be responsible for its excellent catalytic performance. Mou [11] reported that increasing the total N content and the sharing of pyridinic N could enhance the catalytic performance in the oxidation of aromatic hydrocarbons. However, the as prepared UCo@NC catalyst did not conform to this observation (Table 2). Ma et al. [6,50] proposed that the neighboring carbon atoms were activated by graphitic N species to facilitate the adsorption and activation of TBHP in the EB oxidation, and both the activity of the catalyst and yield of the AP were dependent on the content of graphitic N species. Meanwhile, the metallic cobalt species was the active site in our reaction system and the content of metallic cobalt decreased in the order of UCo@NC > MCo@NC > DCo@NC (Table 2). According to the above analysis of the structure–performance relationship of the different catalysts, it is thereby concluded that the superior oxidation activity of UCo@NC was attributed to the synergistic effect between the graphitic nitrogen species and the metallic cobalt species. It is reasonable to deduce that there is an interaction between cobalt and nitrogen, such as the coordination of nitrogen to cobalt, thus leading to the synergistic effect and showing superior catalytic performance in our research.

### 2.3. Oxidation of Other Arylalkanes

In order to study the application scope of UCo@NC catalyst in selective oxidation, various arylalkanes were used as substrates under similar reaction conditions and the results were listed in Table S1. Substituted ethylbenzene containing either electron-donating or electron-withdrawing functional groups exhibited excellent conversions and selectivity to the corresponding ketones (Table S1, entry 1 and 8). Moderate catalytic conversions for the oxidation of bromo-ethylbenzene (85.5%) and nitro-ethylbenzene (66.8%) with selectivity to ketones higher than 90% were obtained (Table S1, entry 2 and 6). Normal propyl-benzene was converted to the corresponding ketone with 43.8% conversion and 74.0% selectivity to the propiophenone (Table S1, entry 3). The conversions of cumene, indene and benzyl methyl ether were 93.8%, 97.8% and 100%, respectively, and good selectivity of the products was also achieved (Table S1, entry 4, 5 and 7). It is pleasing that both the conversions and selectivity for oxidation of diphenylmethane and fluorene were excellent, especially for fluorene, which was oxidized to 9-fluorenone with 100% conversion and 100% selectivity (Table S1, entry 9 and 10). This may be the reason that, in diphenylmethane and the fluorene molecule, the presence of two phenyls could enhance the C-H bond activation and make the oxidation easier. The above results show that the UCo@NC could be used for solvent-free oxidation with a broad range of aromatic hydrocarbons to prepare the corresponding aromatic ketones, with TBHP as the oxidant.

### 2.4. Kinetic Study

**Figure 10:**
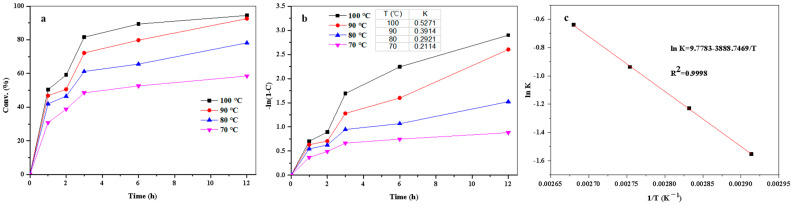
(**a**) EB conversion influenced by the reaction time under different temperatures; (**b**) the logarithm of the content of the substrate profiles obtained at the different reaction temperatures and (**c**) Arrhenius plot of the oxidation of EB over UCo@NC catalyst.

The kinetic experiments were performed in the temperature ranges of 70–100 ℃ over UCo@NC catalyst for EB oxidation. The results display that the conversions of EB obviously increased with the increase in reaction temperatures (Figure 10a), indicating that temperature is an important influencing factor, which is consistent with previous results (Figure 8c). As shown in Figure 10b, the rate constant (K) was obtained from the slope of the linear curve according to the plot of -ln (1-C) versus reaction time at different temperatures, and “C” represents the conversions of EB. Notably, the linear curves were collected within the initial 3 h of the reaction time. Furthermore, the apparent activation energy (E_a_) was calculated from the plot of ln K versus 1/T according to the Arrhenius equation and the value was 32.3 kJ/mol (Figure 10c), which was lower than for some other metal-based catalysts in the EB oxidation reaction. For example, Chaudhary [51] used a polymer-silica hybrid supported bimetallic catalyst (Co-Cu/SAP-Si) for EB oxidation with the calculated E_a_ being 37.02 kJ/mol. The kinetic study showed that the aerobic oxidation of EB was a first-order reaction over CuMgAl-LDH catalyst with 35.2 kJ/mol of E_a_ [52]. Liu [53] reported that EB oxidation with CeVO_4_ had an E_a_ of 32.8 kJ/mol, which was very similar to our result. These results suggest that it is relatively easy for UCo@NC catalyst to trigger EB oxidation.

### 2.5. Gram-Scale Oxidation and Recyclability

A gram-scale oxidation reaction was carried out in order to investigate the practicability of the UCo@NC catalyst for selective oxidation of EB. As can be seen in Table S2, the reaction with the scale-up of EB to 10 mmol (i.e., 1.06 g) could afford AP with 81.0% conversion and 97.6% selectivity in 12 h. When the reaction time was prolonged to 36 h, the yield was increased from 79.1% to 88.1%, with better selectivity (99.5%). The results indicated that the oxidation of EB on a gram-scale proceeded smoothly over a UCo@NC catalyst.

**Figure 11:**
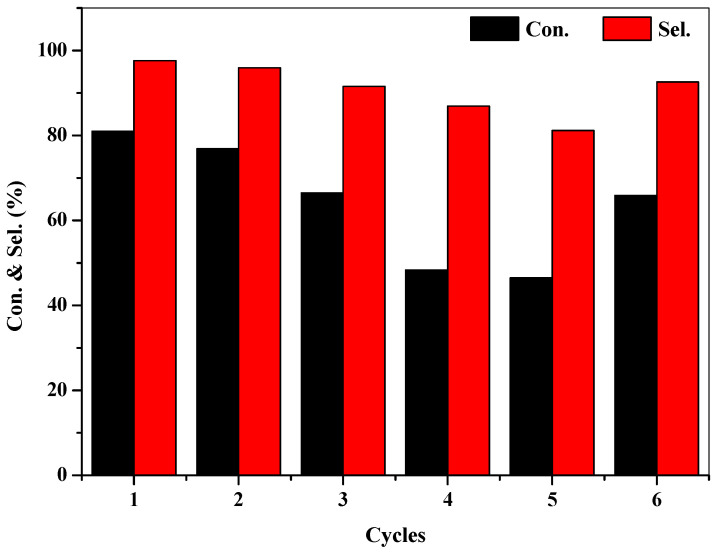
Reusability of UCo@NC catalyst for EB oxidation. Reaction conditions: 10 mmol EB, 50 mg catalyst, 30 mmol TBHP, 100 °C, 12 h.

**Figure 12:**
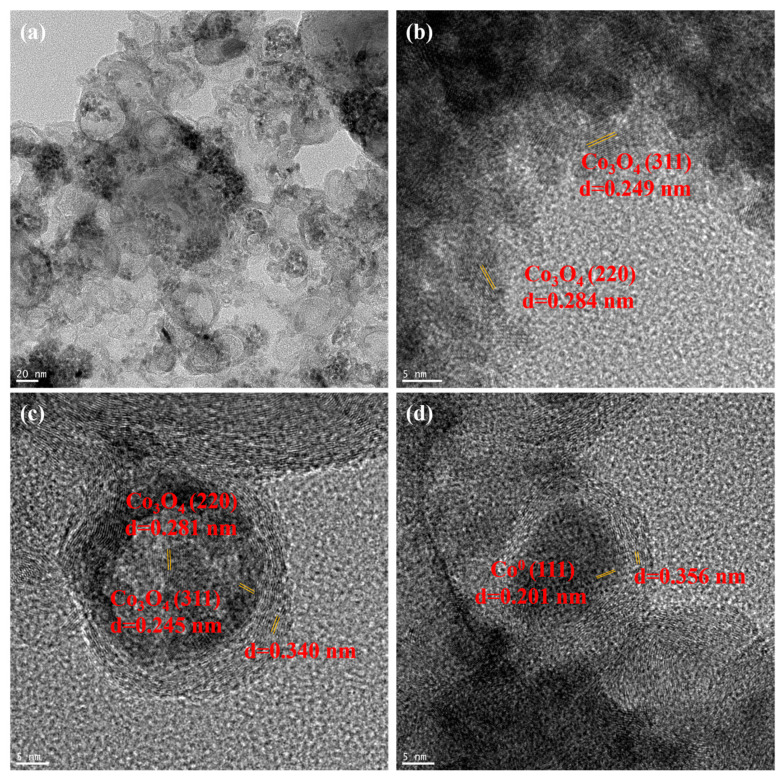
TEM (**a**) and HRTEM (**b**–**d**) images of UCo@NC catalyst after being reused in five runs.

In terms of the reusability of the catalyst, we performed selective oxidation of EB using UCo@NC in six successive runs and the results are compiled in Figure 11. It can be seen that, after 5 cycles, the conversion of EB decreased from 81.0% to 46.5%, while the selectivity to AP could be kept at a relatively high level (81.2%). The results showed that the catalytic performance declines gradually during recycling. The XRD, XPS and TEM characterizations of the UCo@NC catalyst after the fifth cycle were performed in order to reveal the reason for deactivation. As displayed in Figure S7, the distinct characteristic peaks at 31, 37, 45, 59 and 65° were observed in the XRD pattern corresponding to the different planes of the Co_3_O_4_ (JCPDS 43-1003), which indicated that the metallic cobalt NPs were oxidized severely in the process of recycling. The XPS result of UCo@NC after being reused for five runs showed that the content of O increased from 7.99% to 20.16% (Table 2). High resolution Co_2p3/2_ of the reused UCo@NC catalyst is shown in Figure S8a, and the result further confirmed that the metallic cobalt was oxidized to cobalt oxides during the reaction, and the peak corresponding to the metallic cobalt species became much weaker compared with the fresh catalyst (Figure 7). Especially, compared with the fresh UCo@NC sample, the increased intensity of the satellite (786.2 eV) further supported this view, since it came from the Co^2+^ combined with O of Co_3_O_4_ [54,55]. Compared with the fresh catalyst, the percentage of graphitic N increased from 44.3% to 54.5% and it was still the dominant species (Table 2, Figure S8b). More importantly, the conversion of EB was 46.5% in the fifth run, which further indicated that the activity was not only related to the metallic cobalt NPs, but also to the N-doped graphite-like carbon shells, especially the graphitic nitrogen species (Figure S8b). Furthermore, hollow nitrogen-doped carbon shell nanostructures were observed after being reused five times (Figure 12a). It is interesting to find that Yang reported the N, P, and S co-doped hollow carbon shell as a metal-free carbo-catalyst for oxidation of aromatic alkanes [1], which was similar to our catalysts after reuse five times. The crystal lattices of 0.25 nm and 0.28 nm were attributed to the (311) and (220) crystal planes of Co_3_O_4_ NPs [30,56], which were displayed outside (Figure 12b) and inside (Figure 12c) the carbon shell, and such a result was also consistent with the XRD result in Figure S7. Meanwhile, some metallic cobalt NPs were still encapsulated in N-doped graphite-like carbon layers (Figure 12d), suggesting that the carbon layers were stable in the sample (Figure 12c,d). In order to demonstrate the regenerative capability of the UCo@NC catalyst, the catalyst was reheated at 600 °C in N_2_ after the fifth run. It was interesting to observe that the activity of the regenerated catalyst was recovered to a large extent (Figure 11). These results illustrated that the superior catalytic performance of UCo@NC for EB oxidation was attributed to the synergistic effect of the metallic cobalt species and nitrogen-doped graphite-like carbon shells.

### 2.6. Proposed Mechanism

**Scheme 2:**
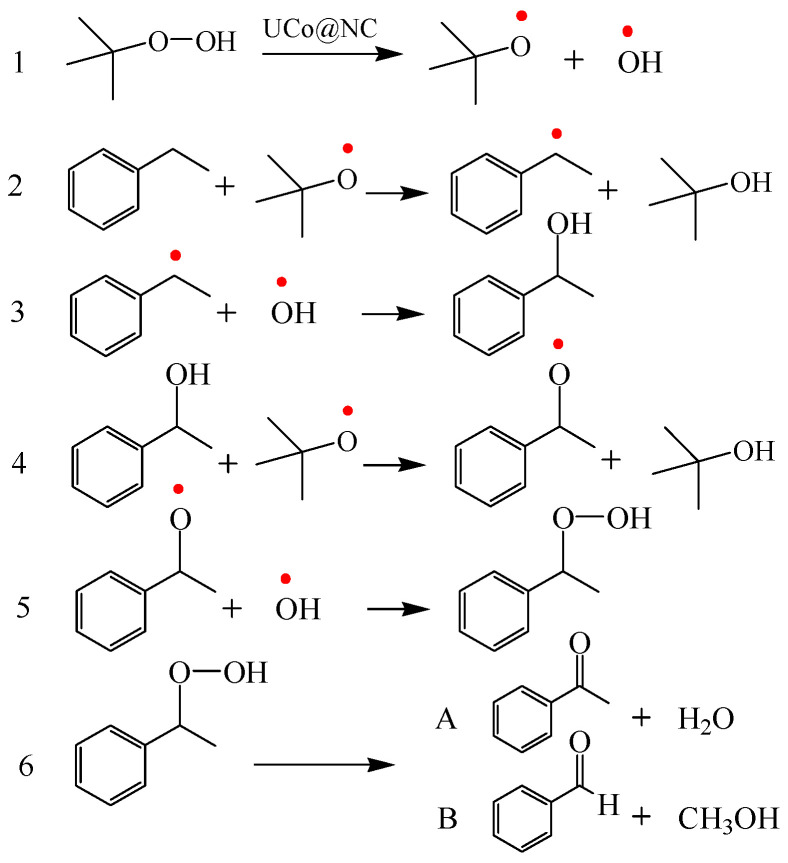
Proposed reaction mechanism of EB oxidation over the UCo@NC catalyst.

As a common oxidant for EB oxidation, TBHP is known to oxidize the substrate via the free radical process [57,58,59]. To test the role of free radicals in the reaction here, *p*-benzoquinone as a radical scavenger was added into the reaction system and the reaction was allowed to proceed for 3 h. In such a case, the conversion of EB was prohibited completely (Table 3, entry 12), which indicated that the oxidation of EB over UCo@NC should also follow the free radical pathway. Further, 1-phenylethanol (PE) was used as substrate instead of EB under the same reaction conditions, and both the conversion and selectivity were 100% (Table S1, entry 11), which indicates that the PE may be the intermediate product. Based on the selective oxidation performance and literature reports, a plausible mechanism for UCo@NC catalyzed EB oxidation is proposed (Scheme 2). Firstly, the O-O bond of the TBHP molecule is activated by the UCo@NC catalyst to generate the tert-butyl oxygen radical and the hydroxyl radical [57]. Then the tert-butyl oxygen radical abstracts an α-H of ethylbenzene to produce the α-ethylbenzene radical, which attacks the hydroxyl radical to form 1-phenylethanol (PE), as has been confirmed by our experiment (Table S1, entry 11). Alternatively, the tert-butyl oxygen radical abstracts a hydrogen atom from the hydroxyl of PE and the ethylbenzene oxygen radical is generated, then the ethylbenzene oxygen radical reacts with the hydroxyl radical to successfully produce ethylbenzene hydroperoxide [58]. Finally, two distinct paths of the carbonyl-forming elimination of ethylbenzene hydroperoxide result in the formation of the major product (AP) and the byproduct (BZ) [59].

### 2.7. Comparison with Other Literature Catalysts

As mentioned previously, harsh reaction conditions, such as high temperatures, high pressures and use of organic solvents, have been generally required for EB oxidation. In this work, the readily available UCo@NC catalyst has achieved the oxidation of ethylbenzene and its derivatives, efficiently using TBHP as an oxidant under a solvent-free condition, therefore showing distinct advantages. Table S3 shows the results obtained by selective catalytic oxidation of ethylbenzene over the UCo@NC catalyst as compared to other heterogeneous Co-based catalysts previously reported in the literature. Very recently, Co/N co-doped hollow mesoporous spheres with carbon/silica binary shell structure catalysts have been reported for EB oxidation using TBHP as oxidant (Table S3, entry 14). However, a very complicated synthetic procedure for the catalyst preparation, highcost of cobalt porphyrin as precursor and high temperature treatment (900 °C) have limited practical industrial application [60]. Similar shortcomings also exist in other reaction systems (Table S3, entry 4 and 5). Atomically dispersed cobalt atoms doped on graphitic carbon nitride (SACo@g-C_3_N_4_) have also been demonstrated as a robust catalyst for EB oxidation using a mixed solution of CH_3_CN/H_2_O as solvent at 60 °C (Table S3, entry 8). Unfortunately, the hazardous oxidant (HSO5-) and CH_3_CN solvent are harmful to the environment, and the 0.1 mmol-scale synthesis did not demonstrate persuasive applicability [61]. Oxygen/air were also used as oxidants for aerobic oxidation of EB to AP, but high temperature/pressure or employment of co-catalysts (e.g., NHPI) were usually unavoidable and both conversion and selectivity were relatively low because oxygen molecules are difficult to become active (Table S3, entry 2, 6, 11–13, 16). In view of the reported cobalt-based catalysts for EB oxidation, it can be concluded that the UCo@NC exhibited a better catalytic activity in most cases. It is worth mentioning that the preparation method of our constructed core-shell catalysts was very simple, effective and did not require any expensive Co/N sources.

## 3. Materials and Methods

### 3.1. Catalysts Preparation

#### 3.1.1. Materials

The following substances were utilized without additional purification: dicyandiamide, melamine, urea, tert-butyl hydroperoxide (TBHP, 70% wt.% in water), cobalt nitrate hexahydrate (Co(NO_3_)_2_·6H_2_O), polyethylene glycol (PEG, Mw = 1000), ethylbenzene (EB), 1-phenylethanol (PE), n-dodecane, ethanol, ethyl acetate, and deionized water, procured from commercial sources.

#### 3.1.2. Synthesis of UCo@NC and DCo@NC

**Scheme 3:**
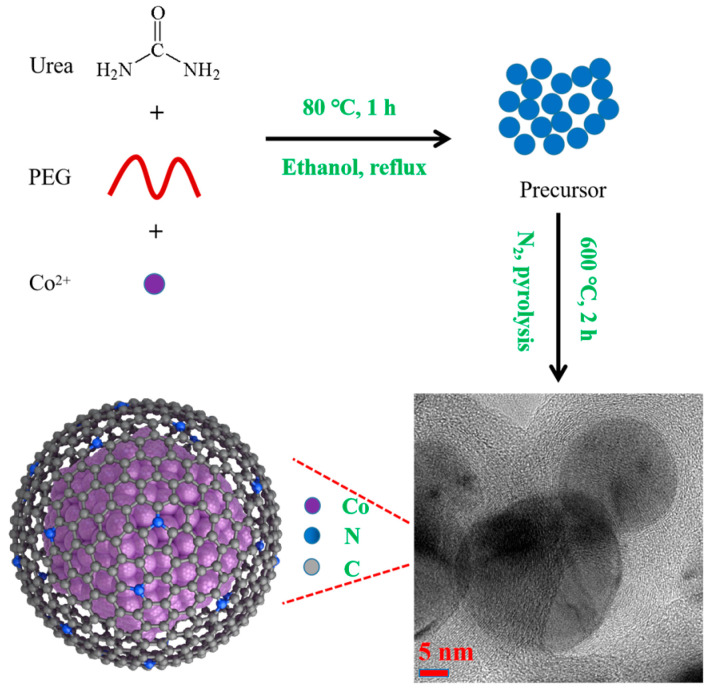
The schematic synthesis of UCo@NC catalyst.

In a typical procedure, 0.8 g of Co(NO_3_)_2_·6H_2_O, 2.0 g urea, 4.0 g of PEG and 25 mL of ethanol were added to a 250 mL of three-necked flask under a magnetic stirring. The resulting mixture was refluxed at 80 °C in an oil (dimethyl silicone oil) bath for 1 h, then the solvent was evaporated at 80 °C, and a paste-like catalyst precursor was obtained. After being cooled down to room temperature, the precursor was transferred into a crucible and heated at 600 °C for 2 h in a tube furnace with a heating rate of 5 °C/min in N_2_ atmosphere (20 mL/min) to obtain a catalyst sample labeled as UCo@NC (the schematic synthetic process is shown in Scheme 3). When the urea was substituted by dicyandiamide, the obtained sample was labeled as DCo@NC.

#### 3.1.3. Synthesis of MCo@NC

Another Co-based catalyst with melamine as nitrogen source was obtained by a similar method as described above, except that 100 mL deionized water was used as the solvent and the oil bath temperature was 110 °C. After the reaction, the deionized water was evaporated at 110 °C until the formation of a paste-like precursor. The resulting sample after pyrolysis was labeled as MCo@NC. The sample Co@C without nitrogen source and sample NC without cobalt salt were prepared using a similar procedure as for the preparation of UCo@NC.

The characterization section and oxidation procedure are provided in the supporting information.

## 4. Conclusions

In summary, the Co@NC series catalysts using different nitrogen sources were synthesized by a one-pot pyrolysis method. The catalysts’ characterizations demonstrated that the metallic Co NPs encapsulated in nitrogen-doped graphite-like carbon layers were successfully constructed. Such core-shell nanostructure catalysts exhibited excellent catalytic performance for the selective oxidation of ethylbenzene with TBHP as oxidant under solvent-free condition. Moreover, the applicability of the catalyst for gram-scale and substrate scope were demonstrated. The investigation indicated that different nitrogen sources could affect the content of metallic cobalt species, as well as the types of nitrogen atoms doped in the carbon layers. The combination of the metallic Co NPs and graphitic N species resulted in an interesting synergistic effect that led to superior catalytic performance. Among the Co@NC samples, a 95.2% conversion of EB and AP selectivity of 96.0% can be achieved under the optimized conditions (2 mmol EB, 10 mg catalyst, TBHP/EB molar ratio of 3, 100 °C, 12 h) by using the UCo@NC catalyst. Besides, a kinetics study of the free radical EB oxidation was performed, and the E_a_ (32.3 KJ/mol) value was obtained, which rationalized the ease of UCo@NC catalyzed oxidation. Therefore, the present study provides easy access to highly active metal-based catalysts, which could be applied in the field of selective oxidation of arylalkanes to aromatic ketones, with excellent efficiency.

## Figures and Tables

**Table 1:** Physico-chemical features of as prepared catalysts.

Catalyst	S_BET_ (m^2^/g)	V (cm^3^/g)	D (nm) ^a^	D_Co_ (nm) ^b^	d_111_ (nm) ^c^
Co@C	221	0.15	2.67	10.9	0.204
DCo@C	366	0.31	3.44	12.7	0.205
UCo@C	80	0.12	6.06	20.7	0.204
MCo@C	362	0.25	2.75	11.0	0.205

^a^ Average pore size. ^b^ The diameter of Co NPs using Scherer equation. ^c^ The d_111_ value was measured according to Bragg’s law.

**Table 2:** The chemical compositions of the as prepared catalysts through XPS analysis.

Catalyst	C (at.%)	O (at.%)	N (at.%)	N_1s_				Co (at.%)	Co_2p3/2_		
				N1	N2	N3	N4		Co^0^	Co^3+^	Co^2+^
Co@C	86.6	10.4	-	-	-	-	-	3.0	29.4	34.1	36.5
DCo@NC	80.2	9.8	8.9	37.0	23.6	29.6	9.7	1.1	12.8	51.1	36.1
UCo@NC	83.6	8.0	7.3	34.0	7.8	44.3	13.9	1.2	25.2	49.4	25.4
MCo@NC	79.6	10.2	9.1	38.0	25.2	27.9	8.9	1.1	18.6	47.3	34.1
UCo@NC ^a^	73.6	20.2	3.0	15.6	21.9	54.5	8.1	3.3	4.6	51.3	44.1

^a^ The catalyst after being reused five times.

**Table 3:** The performance of different catalysts for EB oxidation.

Entry	Catalyst	t (h)	Con. (%)	Sel. (%)
1	blank	3	trace	-
2	NC	3	22.9	68.3
3	Co@C	3	55.0	77.0
4	DCo@NC	3	61.6	84.5
5	UCo@NC	3	81.6	93.2
6	MCo@NC	3	65.3	83.6
7	DCo@NC	12	90.2	95.4
8	UCo@NC	12	95.2	96.0
9	MCo@NC	12	90.3	95.3
10 ^a^	UCo@NC	12	94.0	96.3
11 ^b^	UCo@NC	24	90.6	95.8
12 ^c^	UCo@NC	3	trace	-

Reaction conditions: 2 mmol EB, 6 mmol TBHP (70 wt.% in water), 10 mg cat., 100 °C. ^a^ 90 °C. ^b^ 80 °C. ^c^ *p*-benzoquinone, at a concentration of one mmol, was introduced into the reaction system.

## Data Availability

Data are contained within the article.

## Supplementary Materials

The following supporting information can be downloaded at: https://www.mdpi.com/article/10.3390/molecules29010065/s1, Figure S1: XRD pattern of the Co@C catalyst; Figure S2: TEM and HRTEM images of Co@C catalyst. The inset in (a) is the corresponding particle size distribution of Co NPs; Figure S3: XPS survey spectra of the different catalysts; Figure S4: Schematic diagram of four nitrogen types; Figure S5: The percentage of surface nitrogen species on the different catalysts; Figure S6: The mass spectrum of the products in the system of catalytic ethylbenzene oxidation: (a) AP; (b) PE; (c) BZ; Figure S7: XRD pattern of UCo@NC catalyst after being reused five runs; Figure S8: Co_2p3/2_ and N_1s_ XPS spectra of UCo@NC catalyst after being reused five runs; Table S1: Catalytic oxidation several of substrates by UCo@NC; Table S2: Gram-scale selective oxidation of EB; Table S3: Comparison of ethylbenzene oxidation with different Co-based catalysts. Refs. [62,63] are cited in the Supplementary Materials.
