# Supplementary material for: Cobalt Encapsulated in Nitrogen-Doped Graphite-like Shells as Efficient Catalyst for Selective Oxidation of Arylalkanes
Source: Molecules. 2023 Dec 21;29(1):65. doi: 10.3390/molecules29010065 (PMC10779642; doi:10.3390/molecules29010065)
Supplement: Supplementary file 1 [file molecules-29-00065-s001.zip › molecules-2685865-supplementary.pdf]

molecules

Supplementary Materials

Cobalt Encapsulated in Nitrogen-Doped Graphite-like Shells as Efficient Catalyst for Selective Oxidation of Arylalkanes

Shuo Li [1,2], Shafqat Ali [3], Zareen Zuhra [3], Huahuai Shen [1], Jiaxiang Qiu [3], Yanbin Zeng [3], Ke Zheng [1], Xiaoxia Wang [1,*], Guanqun Xie [3,*] and Shujiang Ding [2]

[1] School of Materials Science and Engineering, Dongguan University of Technology, Dongguan 523808, China
[2] School of Chemistry, Xi'an Key Laboratory of Sustainable Energy Materials Chemistry, Xi'an Jiaotong University, Xi'an 710049, China
[3] School of Environment and Civil Engineering, Dongguan University of Technology, Dongguan 523808, China
* Correspondence: wangxx@dgut.edu.cn (X.W.); gqxie@dgut.edu.cn (G.X.)

Catalyst Characterization

X-ray diffraction (XRD) was performed on a Rigaku SmartLab 9 Kw from 10-80° at 5°/min. The basic physical properties of the catalysts were determined by N_2 adsorption/desorption isotherms performed at -196 °C by using a Micromeritics ASAP2460 surface area and porosity analyzer. The specific surface areas (S_{BET}) were calculated according to the BET equation, the pore volume (V) was calculated by the N_2 adsorption amount at P/P_0 of 0.99, and the pore size distribution was obtained by the BJH method using the desorption branch of the isotherm. X-ray photoelectron spectroscopy (XPS) was performed using a Thermo Scientific K-Alpha spectrometer equipped with Al Kα (1486.6 eV) radiation as the excitation source. The operating pressure in the analysis chamber was kept below 2×10^{-7} mbar. All the binding energy (BE) values were calibrated to the C_{1s} electron binding energy at 284.8 eV. The catalysts for Raman spectra were obtained from a Witec alpha $300M^+$ Raman spectrometer equipped with a 532 nm laser excitation at room temperature. The morphologies and structures of catalysts were performed by transmission electron microscopy (TEM) with a FEI Talos F200S electron microscope (FEI Co., USA) operating at a voltage of 200 kV under high vacuum conditions.

Oxidation Reaction

Liquid phase selective oxidation of arylalkanes to the corresponding ketones without solvent was conducted in this study. The oxidation reaction was carried out in a 15 mL glass tube sealed with teflon lid. Typically, 2 mmol EB, 6 mmol TBHP (70 wt% in H_2O) and 10 mg catalyst were added to the tube heated in an oil bath for 12 h with continuous magnetic stirring at 100 °C. When the reaction tube was cooled down to room temperature, 0.1 mL n-dodecane was added as an internal standard. The reaction solution was quantitatively analyzed by Shimadzu GC-2010 gas chromatography (GC) equipped with a flame ionization detector. The product identity was confirmed by GC-MS (Shimadzu QP2010). The catalyst was recovered by centrifugation, washing three times with ethyl acetate and drying at 80 °C for 10 h and then used for the next run.

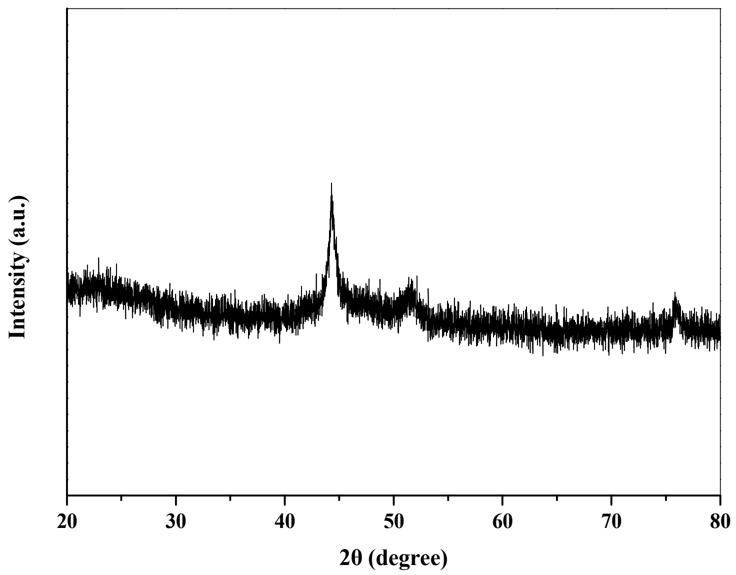

Figure S1. XRD pattern of the Co@C catalyst.

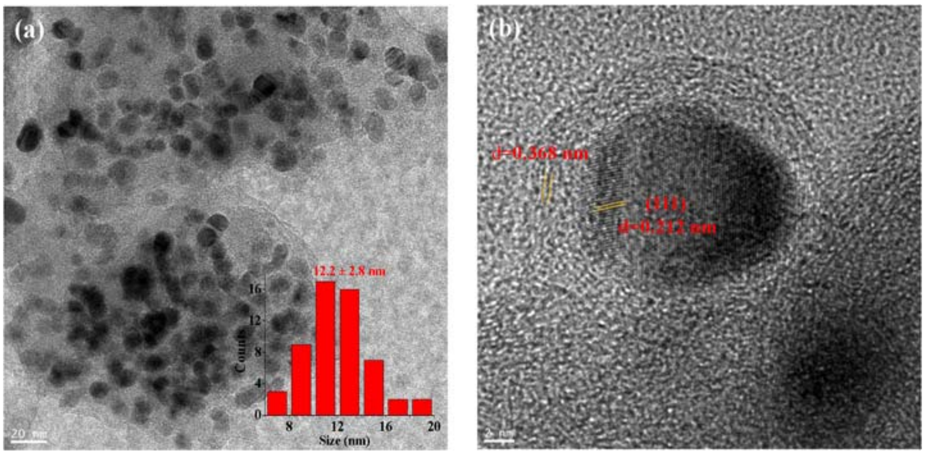

Figure S2. TEM and HRTEM images of Co@C catalyst. The inset in (a) is the corresponding particle size distribution of Co NPs.

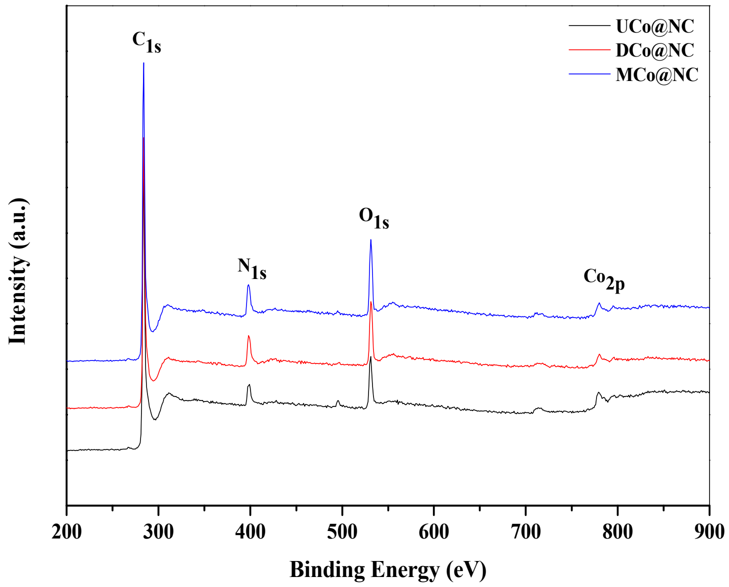

Figure S3. XPS survey spectra of the different catalysts.

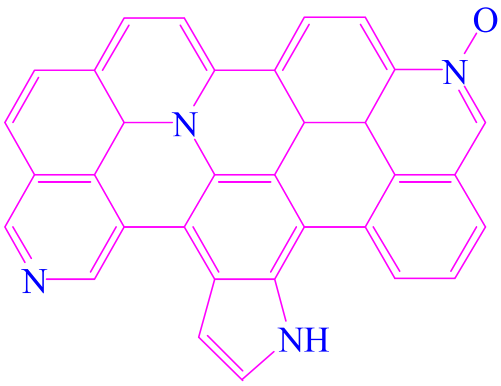

Figure S4. Schematic diagram of four nitrogen types.

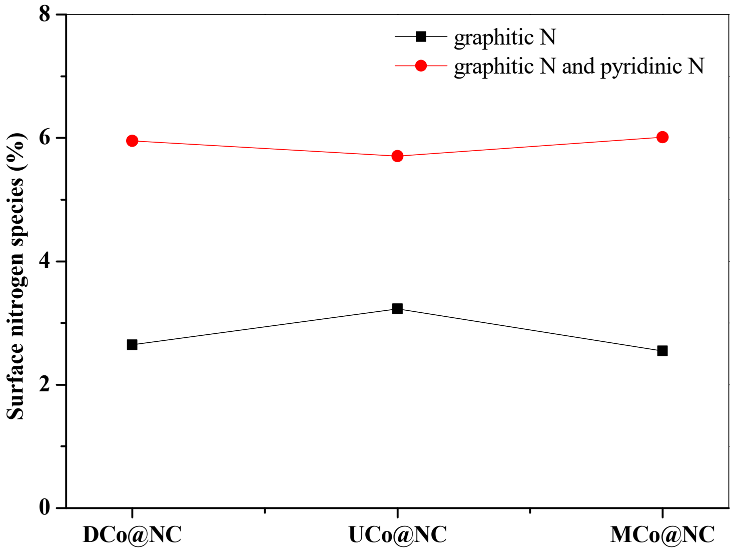

Figure S5. The percentage of surface nitrogen species on the different catalysts.

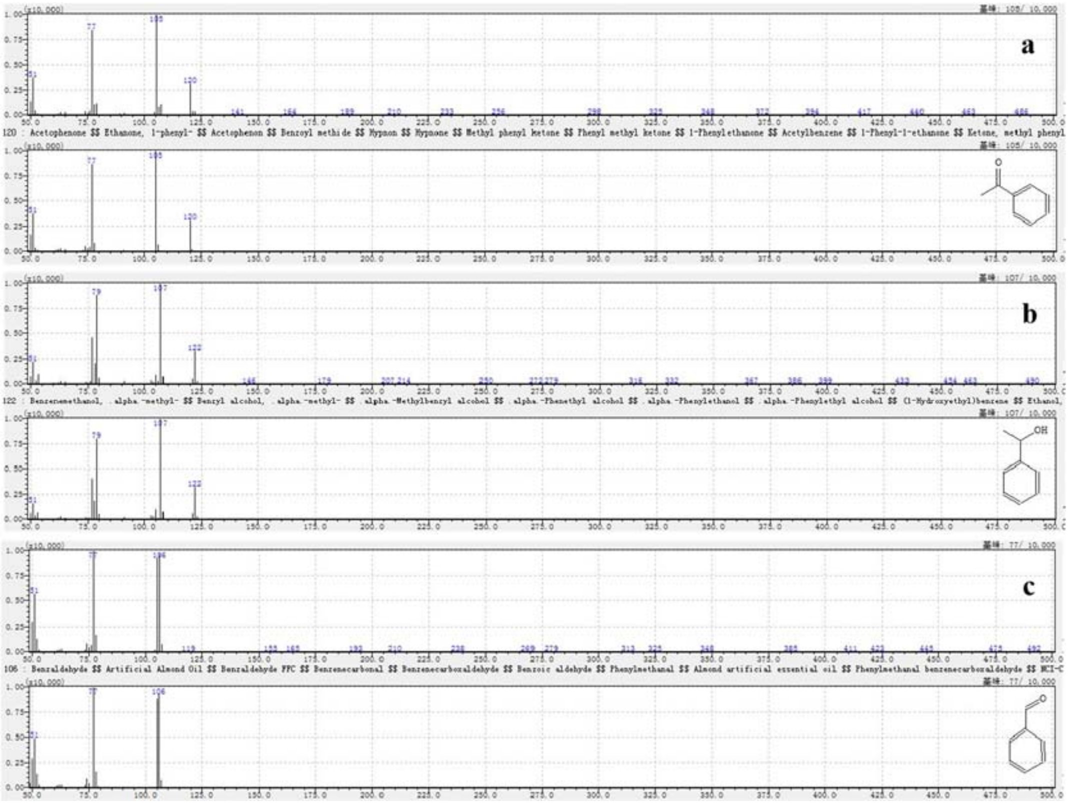

Figure S6. The mass spectrum of the products in the system of catalytic ethylbenzene oxidation: (a) AP; (b) PE; (c) BZ.

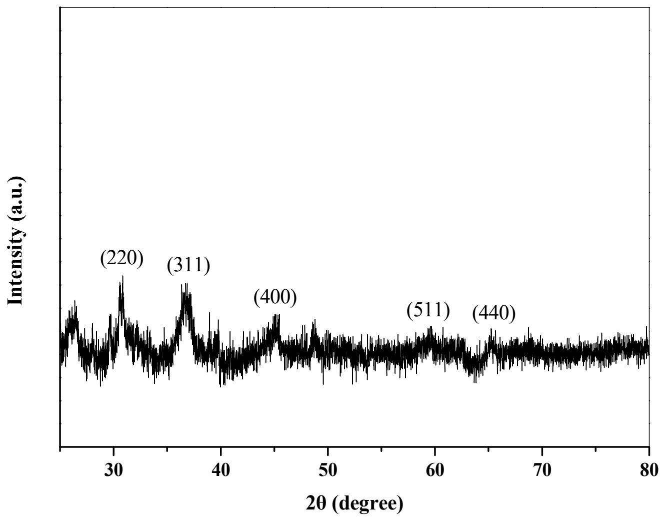

Figure S7. XRD pattern of UCo@NC catalyst after being reused five runs.

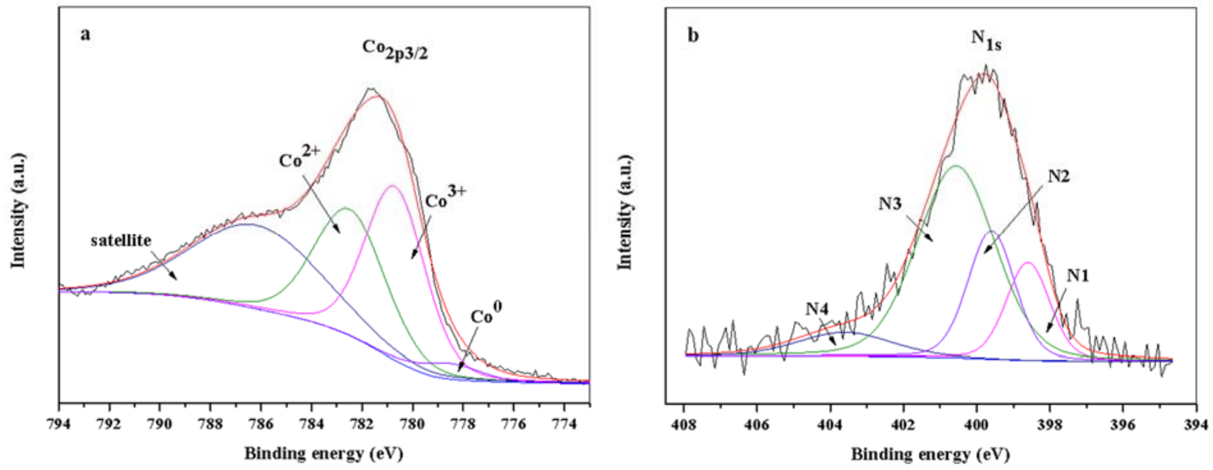

Figure S8. $Co_{2p3/2}$ and N_{1s} XPS spectra of UCo@NC catalyst after being reused five runs.

Table S1. Catalytic oxidation of several substrates by UCo@NC.

Entry	Substrate	Product	Con. (%)	Sel. (%)
1	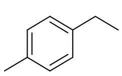	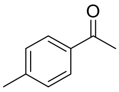	82.2	95.2
2	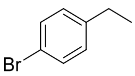	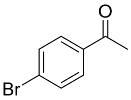	85.5	97.0
3	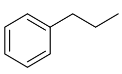	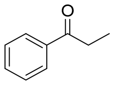	43.8	74.0
4	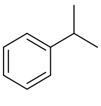	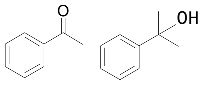	93.8	36.1, 63.9
5	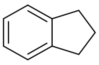	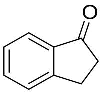	97.8	90.0
6	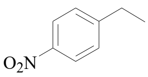	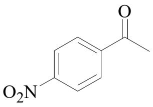	66.8	90.2
7	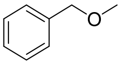	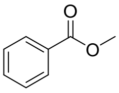	100	81.4
8	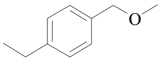	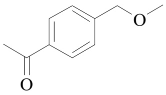	93.5	97.5
9	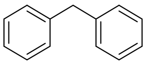	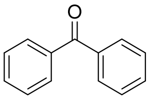	94.6	100
10	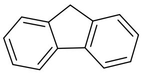	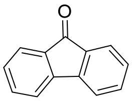	100	100
11	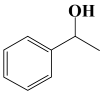	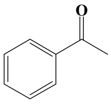	100	100

Reaction conditions: 2 mmol substrate, 6 mmol TBHP (70 wt% in water), 10 mg cat., 100 °C, 6 h.

Table S2. Gram-scale selective oxidation of EB.

Time (h)	Con. (%)	Sel. (%)	Yield (%)
12	81.0	97.6	79.1
24	86.4	98.4	85.0
36	88.6	99.5	88.1

Reaction conditions: 10 mmol EB, 30 mmol TBHP (70 wt% in water), 50 mg cat., 100 °C.

Table S3. Comparison of ethylbenzene oxidation with different Co-based catalysts.

Entry	Catalyst	Oxidant	Solvent	P (Mpa)	T (°C)	t (h)	Con. (%)	Sel. (%)	Ref.
1	Al_2O_3@CoCuAl-MMO	TBHP	-	-	120	12	92.8	89.4	[1]
2	Co@GCNs	O_2	-	0.8	120	5	68.1	93.2	[2]
3	Co-N/C-700	TBHP	H_2O	-	80	6	88	89	[3]
4	Co-N/C-S-H	TBHP	H_2O	-	80	6	96	99	[4]
5	Co-N-C/PCMK	TBHP	H_2O	-	80	12	96	99	[5]
6	Co/rGo/NHPI	O_2	CH_3CN	0.3	120	2	84.1	96.2	[6]
7	Cu-Co/SA-2-AMPS-SiO_2	TBHP	-	-	100	6	83.7	87.2	[7]
8	SACo@g-C_3N_4	HSO_5^-	CH_3CN/H_2O	-	60	15	97.5	95.6	[8]
9	Co-GS-900	TBHP	H_2O	-	80	8	91	98	[9]
10	Co SACs	Air	-	-	120	24	47	97	[10]
11	Co_2Fe-LDH/NHPI	O_2	$C_7H_5F_3$	-	80	10	97.6	94.9	[11]
12	Co/N-CFF@TiO_2-SiO_2	O_2	-	2	130	12	25	88	[12]
13	Co_3O_4@GNC-B	Air	-	2	140	4	65.8	72.6	[13]
14	900-Co-N_x-C@C/mSiO_2	TBHP	H_2O	-	80	12	95.9	99.3	[14]
15	Co/AC-salen-400	TBHP	CH_3CN	-	80	4	47.9	83.5	[15]
16	mCo_3O_4-350	O_2	-	1	120	7	42	90	[16]
17	UCo@NC	TBHP	-	-	100	6	89.4	95.6	Herein
18	UCo@NC	TBHP	-	-	100	12	95.2	96.0	Herein

References

1. Xie, R.; Fan, G.; Yang, L.; Li, F. Hierarchical flower-like Co-Cu mixed metal oxide microspheres as highly efficient catalysts for selective oxidation of ethylbenzene. *Chem. Eng. J.* **2016**, 288, 169-178.
2. Lin, X.; Nie, Z.; Zhang, L.; Mei, S.; Chen, Y.; Zhang, B.; Zhu, R.; Liu, Z. Nitrogen-doped carbon nanotubes encapsulate cobalt nanoparticles as efficient catalysts for aerobic and solvent-free selective oxidation of hydrocarbons. *Green Chem.* **2017**, 19, 2164-2173.
3. Chen, Y.; Jie, S.; Yang, C.; Liu, Z. Active and efficient Co-N/C catalysts derived from cobalt porphyrin for selective oxidation of alkylaromatics. *Appl. Surf. Sci.* **2017**, 419, 98-106.
4. Jie, S.; Lin, X.; Chen, Q.; Zhu, R.; Zhang, L.; Zhang, B.; Liu, Z. Montmorillonite-assisted synthesis of cobalt-nitrogen-doped carbon nanosheets for high-performance selective oxidation of alkyl aromatics. *Appl. Surf. Sci.* **2018**, 456, 951-958.
5. Zhang, L.; Jie, S.; Cheng, N.; Liu, Z. Solvent-free melting-assisted pyrolysis strategy applied on the Co/N codoped porous carbon catalyst. *ACS Sustainable Chem. Eng.* **2019**, 7, 19474-19482.
6. Gao, L.; Zhuge, W.; Feng, X.; Sun, W.; Sun, X.; Zheng, G. Co/rGO synthesized via the alcohol-thermal method as a heterogeneous catalyst for the highly efficient oxidation of ethylbenzene with oxygen. *New J. Chem.* **2019**, 43, 8189-8194.
7. Chaudhary, V.; Sharma, S. Study of ethylbenzene oxidation over polymer-silica hybrid supported Co (II) and Cu (II) complexes. *Catal. Today* **2021**, 375, 601-613.
8. Li, J.; Zhao, S.; Yang, S.; Wang, S.; Sun, H.; Jiang, S.; Johannessen, B.; Liu, S. Atomically dispersed cobalt on graphitic carbon nitride as a robust catalyst for selective oxidation of ethylbenzene by peroxymonosulfate. *J. Mater. Chem. A* **2021**, 9, 3029-3035.
9. Xia, M.; Huang, H.; Zhang, X.; Wei, Q.; Xie, Z. Single-atom cobalt-fused biomolecule-derived nitrogen-doped carbon nanosheets for selective oxidation reactions. *Phys. Chem. Chem. Phys.* **2021**, 23, 14276-14283.

10. Xiong, Y.; Sun, W.; Han, Y.; Xin, P.; Zheng, X.; Yan, W.; Dong, J.; Zhang, J.; Wang, D.; Li, Y. Cobalt single atom site catalysts with ultrahigh metal loading for enhanced aerobic oxidation of ethylbenzene. *Nano Res.* **2021**, 14, 2418-2423.
11. Zhou, W.; Lu, W.; Sun, Z.; Qian, J.; He, M.; Chen, Q.; Sun, S. Fe assisted Co-containing hydrotalcites catalyst for efficient aerobic oxidation of ethylbenzene to acetophenone. *Appl. Catal. A Gen.* **2021**, 624, 118322.
12. Hosseini, S.; Ghiaci, M.; Kulinich, S.; Wunderlich, W.; Ghaziaskar, H.; Koupaei, A. Ethyl benzene oxidation under aerobic conditions using cobalt oxide imbedded in nitrogen-doped carbon fiber felt wrapped by spiral TiO_2-SiO_2. *Appl. Catal. A Gen.* **2022**, 630, 118456.
13. Pendem, S.; Singuru, R.; Sarkar, C.; Joseph, B.; Lee, J.; Shinde, D.; Lai, Z.; Mondal, J. Zeolitic imidazolate framework-mediated synthesis of Co_3O_4 nanoparticles encapsulated in N-doped graphitic carbon as an efficient catalyst for selective oxidation of hydrocarbons. *ACS Appl. Nano Mater.* **2018**, 1, 4836-4851.
14. Xiang, G.; Zhang, L.; Yi, C.; Liu, Z. One-pot pyrolysis method to fabricate Co/N co-doped hollow mesoporous spheres with carbon/silica binary shells for selective oxidation of arylalkanes. *Appl. Surf. Sci.* **2022**, 577, 151829.
15. Nakatsuka, K.; Yoshii, T.; Kuwahara, Y.; Mori, K.; Yamashita, H. Controlled synthesis of carbon-supported Co catalysts from single-sites to nanoparticles: characterization of the structural transformation and investigation of their oxidation catalysis. *Phys. Chem. Chem. Phys.* **2017**, 19, 4967-4974.
16. Liu, Y.; Zheng, Y.; Feng, D.; Zhang, L.; Zhang, L.; Song, X.; Qiao, Z. Efficient selective oxidation of aromatic alkanes by double cobalt active sites over oxygen vacancy-rich mesoporous Co_3O_4. *Angew. Chem. Int. Ed.* **2023**, 62, e202306261.
